# Surgical patients’ use of, and attitudes towards, the internet for e-patient activities in Germany and Oman

**DOI:** 10.1016/j.amsu.2020.05.022

**Published:** 2020-06-03

**Authors:** Ken Masters, Teresa Loda, Rashid Al-Abri, Jonas Johannink, Anne Herrmann-Werner

**Affiliations:** a Medical Education and Informatics Department, College of Medicine and Health Sciences, Sultan Qaboos University, Oman; b Medical Department VI/Psychosomatic Medicine and Psychotherapy, University Hospital Tübingen, Tübingen, Germany; c Department of Surgery and Transplantation, University Hospital Tübingen, Tübingen, Germany

**Keywords:** e-patient, Surgeon-patient relationship, Communication

## Abstract

**Introduction:**

E-patient activities are known to impact upon the patient-doctor relationship and on surgical outcomes. In Oman, there is no published information about the e-patient. The aim of this study, conducted at two surgical sites, was to investigate surgical e-patients’ use of, and attitudes towards, the Internet, and the possible impact on the delivery of healthcare.

**Materials and methods:**

A convenience sample of 83 German and 93 Omani patients at the two surgical sites were given an in-house paper-based questionnaire, based on e-patient activities described in the literature. Descriptive statistics like means, standard deviations and frequencies were calculated.

**Results:**

There were many similarities in usage and attitudes. Omani patients showed much greater knowledge and usage of sites and apps, used the Internet more for health-related activities (26.9% vs. 12.0%), and had a greater proportion of their physician encounters through email (10.0% vs. 4.0%) and social media (15.2% vs. 1.8%). More Omani patients brought information from the Internet than German patients (13.5% vs. 6.0%). Patients from both countries were generally positive about bringing material from the Internet to the consultation.

**Discussion and conclusion:**

Both sites indicated typical e-patient activity and attitudes as described in the literature. Age and type of condition (chronic vs. acute) may explain the differences to some extent, but this was not consistent. Socio-cultural differences between the two countries may have a great influence on the usage.

## Highlights

• Although both sites indicated typical e-patient activity and attitudes, the Omanis had greater use than the Germans.
• This was evident in overall Internet use for health-related activities and for social media interaction with their surgeons.
• All were positive about bringing Internet material to consultations, potentially impacting on doctor-patient relationships.

## 1 Introduction

### 1.1 Background and literature

In the 1990s, Bill Gates foresaw patients’ use of the Internet to find health-related information, communicate with other patients and health professionals, and access their Electronic Medical Records (EMRs) [1]. Today, we speak of “e-patients”, first described by Tom Ferguson in 2007 as patients who are “equipped, enabled, empowered and engaged in their health and health care decisions” [2] and who use “the Internet to gather information about a medical condition of particular interest to them” [2].

E-patient activities closely resemble Gates’ predictions, and usually include searching for medical and health-related information on the Internet [[3], [4], [5], [6], [7], [8], [9], [10]], joining patient discussion groups [3,5,[8], [9], [10], [11], [12], [13]], emailing their physicians [3,5,9,10,12,[14], [15], [16], [17]], accessing EMRs [3,10,16,18], and other health-related activities [3,4,[6], [7], [8],^10^,^12^,^13^,^16^,^19^,^20^].

While patient engagement is generally encouraged [[21], [22], [23]], e-patient activities have a significant impact on the patient-doctor relationship; this impact can be positive or negative, and can impact positively or negatively on health outcomes and treatment [4,21,[24], [25], [26], [27], [28], [29], [30], [31]].

Doctors' attitudes towards patients' using the Internet directly influences that usage, and impacts upon the quality of material found, and the patient-doctor relationship [6,21,24,[32], [33], [34]]. Contrastingly, about 65% of e-patients *do not* share Internet-based information with their doctors, usually because of their doctors’ negative attitudes towards the Internet [24,25,29,30]. This lack of openness could potentially harm patient-doctor communication and the relationship as a whole.

E-patients undergoing surgery consult the Internet pre- and post-surgery, and approximately 20% of e-patients consult sites recommended by their surgeon or doctor, rating those sites higher in quality than other sites they have consulted [6]; more informed patients have experienced less post-operative pain than uninformed patients [35]. E-patients also use the Internet to research their surgeons [6,31,36].

### 1.2 Setting

In Germany and Oman, approximately 90% and 80% of the population accesses the Internet, respectively [37,38]. Based on previous research, there are at least 49 million e-patients in Germany [39,40]. This represents a potentially great impact on the patient-doctor relationship.

#### 1.2.1 Rationale

There are currently no figures for e-patients in Oman, and reports on Omani e-patient activities rely on extrapolation from other countries [3], a practice with obvious weaknesses [41]. While Internet adoption patterns in Oman may resemble other countries' patterns, definitive data are required for appropriate responses to be generated. This need is amplified by a recent study on surgeons’ use of the Internet in Oman [42] showing some distinctive usage traits, notably in social media for patient-surgeon communication.

### 1.3 Aim of this study

This study focusses on surgical e-patients’ use of, and attitudes towards, the Internet in Germany and Oman. The aim is to gauge patients’ Internet usage patterns, attitudes towards e-patient activities, and the possible impact on the delivery of healthcare.

## 2 Material and methods

### 2.1 Variables

The main independent variable is the patient's country of residence (Germany or Oman); further independent variables derived from the literature indicating possible predictors of e-patients’ activities and attitudes included age, gender, amount of Internet usage, work-related time on the Internet, and whether suffering from chronic or acute conditions [10,12,20,23].

The dependent variables were made up from the following:

• the patients' knowledge and typical e-patient activities as described in the literature in the Introduction above. As we felt that “searching online” would be too broad, we made a finer distinction about the type of sites from which they would draw their information.
• As we wanted to know if their not using a type of site was because of lack of knowledge, before asking about usage, we asked if they knew of the type of site.
• As the literature has long examined the impact of doctors' recommending specific sites to their patients,[6,32,[43], [44], [45], [46]] we added two questions about doctors' recommendations of sites and apps.
• As the literature indicates that doctors are using social media to communicate with their patients [47], we added two questions about that.
• As neither of the medical centres permitted their patients to view their EMRs online, we did not ask questions about that activity.
• Patients' perceptions of doctors' attitudes were based on questions from a study of German doctors performed by Moick and Terlutter [48].

The work has been reported in line with the STROCSS criteria [49].

### 2.2 Questionnaire design

The consent form and questionnaire (Appendix 1) was constructed in English and translated into Arabic and German. In German, ethics approval was obtained from the University of Tübingen Medical Ethics Committee (001/2018BO2); in Oman, from the Sultan Qaboos University, College of Medicine and Health Sciences Research Ethics Committee (MREC#1179).

Hours per day of Internet usage were in intervals of 2 h, ranging from 0 to greater than 10; Percentages of work-related time, email and social media interactions with their doctor, bringing material from the internet to the consultation, and doctors’ recommendations of sites and apps were in intervals of 5%, ranging from 0 to 100. Following Moick and Terlutter [48], attitudes were on a Likert scale of 1 (absolutely disagree) to 7 (absolutely agree).

### 2.3 Questionnaire delivery

The information sheet and consent form contained the title, description of the research project, names and contact details of the researchers, a statement about risks, confidentiality, storage of information (256-bit encryption), the voluntary nature of the participation, and permission to retain (or obtain) a copy of the informed consent form. All patients signed the consent form. After completion and collection, the consent form was separated from the questionnaire, and housed in a separate location.

In Germany, the consent form and questionnaire (German) were delivered to patients attending the General Surgery clinic at University Hospital XX. Patient participation was supervised by one of the co-investigators (XX).

In Oman, the consent form and questionnaire (English and Arabic) were delivered on paper to patients in Surgery Clinics (ENT, General surgery and Urology) at Sultan Qaboos University. Patient participation was supervised by one of the co-investigators (RA).

The consent form and questionnaire were delivered on paper to the patients during March–July 2018. All adult patients in the clinics were eligible, and were invited by the supervising co-investigator, and the forms were completed in the clinics and collected by the co-investigator. We aimed for 80–90 patients which would be large enough to give meaningful statistics, but not disrupt the clinic's day-to-day functions. As a result, we distributed 100 questionnaires at each of the sites.

All questionnaires were analysed. Data were captured into SPSS (Ver 25) and MS-Excel 2016. Main data analysis methods were used. Descriptive statistics like means, standard deviations and frequencies were calculated. Data were normally distributed as tested by the Kolmogorov-Smirnov-Test, and missing values were replaced by means. Pearson correlations tested for correlations between variables. Differences between the German and Omani patients were determined by chi-squared tests, *t*-test for independent samples, Wilcoxon-Test and ANOVA tests. Statistical significance was taken at p < .05. (For the sake of brevity, the Results speak of “German” and the “Omani” patients, although these do not refer to nationality, but location at the time of study).

## 3 Results

A total of 83 German and 93 Omani patients completed the survey form, giving an 83% and 93% response rate, respectively (88% overall).

We established the patients’ age, gender and whether their visit that day was because of a chronic or acute condition. Table 1 summarises these results.

**Table 1:** Patients’ Age (years), Gender (female/male/prefer not to say), Acute or Chronic condition visit, by country.

	Germany	Oman	Overall	Statistics
n	83	93	176	F (1, 174) = 53.90; *P* < .001
Age Mean	50.72	36.08	42.98	
Age SD	16.675	10.544	15.078	
Age Range	17–85	20–85	17–85	
Age Quartile 1	38.0	30.0	32.0	
Age Median	52.0	34.0	39.5	
Age Quartile 3	62.0	41.5	53.5	
Female (n/%)	49 (59.0)	48 (53.3)	97 (56.1)	χ^2^(1) = 0.57, *P* = .450
Male (n/%)	34 (41.0)	42 (45.2)	76 (43.9)	
Unknown gender (n/%)	0	3 (3.2)	3 (1.7)	
Chronic (n/%)	28 (33.7)	40 (43.0)	68	χ^2^(2) = 37.05, *P* < .001
Acute (n/%)	14 (16.9)	49 (52.7)	63	
Both (n/%)	28 (33.7)	4 (4.3)	32	

The Omanis (M = 3.43, SD = 1.51) spent significantly (F=(1, 172) = 45.2, p < .001) more hours on the Internet than the Germans (M = 2.10, SD = 1.01, overall: M = 2.81, SD = 1.46). There were significant inverse correlations for age and hours spent on the Internet for both Omanis (r = −0.223, p = .031) and Germans (r = − 0.619, p < .001).

### 3.1 Internet sites and app knowledge and usage

We measured the patients’ knowledge and usage of types of Internet sites (See Table 2, Table 3).

**Table 2:** Patients’ knowledge of types of Internet sites, by country.

Site	Germany (N = 83)		Oman (N = 93)		TOTAL (N = 176)		χ^*2*^
	n	%	n	%	n	%	
Books	21	25.3	60	64.5	81	46.8	χ^2^(1) = 25.29, *P* < .001
Videos	38	45.8	80	86.0	118	68.2	χ^2^(1) = 29.43, *P* < .001
General references	41	49.4	39	41.9	80	46.2	χ^2^(1) = 1.5, *P* = .22
Networking sites	19	22.9	27	29.0	46	26.6	χ^2^(1) = 0.62, *P* = .43
Official/Institutional	14	16.9	11	11.8	25	14.5	χ^2^(1) = 1.12, *P* = .29
Databases	6	7.2	22	23.7	28	16.2	χ^2^(1) = 8.28, *P* = .004
Journals	5	6.0	16	17.2	21	12.1	χ^2^(1) = 4.84, *P* = .028
Magazines	37	44.6	16	17.2	53	30.6	χ^2^(1) = 17.074, *P* < .001

**Table 3:** Patients’ use of types of Internet sites at least once per month, by country.

Site	Germany (N = 83)		Oman (N = 93)		TOTAL (N = 173)		χ^*2*^
	N	%	n	%	n	%	
Books	15	18.1	46	49.5	62	35.8	χ^2^(1) = 18.9, *P* < .001
Videos	27	32.5	69	74.2	95	54.9	χ^2^(1) = 26.92, *P* < .001
General references	25	30.1	22	23.7	47	27.1	χ^2^(1) = 1.253, *P* = .26
Networking sites	19	22.9	20	21.5	39	22.5	χ^2^(1) = .124, *P* = .73
Official/Institutional	4	4.8	8	8.6	12	6.9	χ^2^(1) = 8.64, *P* = .35
Databases	7	8.4	14	15.1	21	12.1	χ^2^(1) = 1.602, *P* = .21
Journals	3	3.6	10	10.8	13	7.5	χ^2^(1) = 3.03, *P* = .082
Magazines	21	25.3	9	9.7	30	17.3	χ^2^(1) = 8.48, *P* = .004

The Omani patients had more knowledge about health-related databases, books, journals and videos, while the German participants knew more about magazines.

Expectedly, usage patterns were similar to knowledge patterns. Videos was the most popular category among both groups.

We measured the patients’ knowledge and usage of types of apps (See Table 4, Table 5).

**Table 4:** Patients’ knowledge of health apps, by country.

App	Germany (N = 83)		Oman (N = 93)		TOTAL (N = 176)		χ^*2*^
	n	%	n	%	n	%	
Monitoring	15	18.1	47	50.5	62	36.0	χ^2^(1) = 18.44, *P* < .001
Information	22	26.5	42	45.2	64	37.2	χ^2^(1) = 5.48, *P* = .019
Tools	21	25.3	34	36.6	55	32.0	χ^2^(1) = 1.96 *P* = .16
Videos	24	28.9	78	73.9	102	59.3	χ^2^(1) = 50.64, *P* < .001

**Table 5:** Patients’ use of health apps at least once per month, by country.

App	Germany (N = 83)		Oman (N = 93)		TOTAL (N = 176)		χ^*2*^
	n	%	n	%	n	%	
Monitoring	5	6	27	29.0	32	18.6	χ^2^(1) = 14.54, *P* < .001
Information	10	12	33	35.5	43	25.0	χ^2^(1) = 11.87, *P* = .001
Tools	10	12	15	16.1	25	14.5	χ^2^(1) = 0.41, *P* = .52
Videos	18	21.7	64	68.8	82	47.7	χ^2^(1) = 36.28, *P* < .001

The figures show generally greater knowledge of apps by the Omanis than the Germans. Videos, again, was the most common category among both groups.

Again, usage patterns were similar to knowledge patterns, and videos were also the most popular application.

### 3.2 Other e-patient activities

Of these patients, 35/73 (47.95%) Germans and 32/91 (35.16%) Omanis had heard of the term “e-patient”, and 38/78 (48.72%) Germans and 39/93 (41.94%) Omanis communicated with their doctors via email or social media.

In addition, we found some significant differences between the countries, including important differences in the methods of electronic communication.

• 26.9% of the Omanis and 12.0% of the Germans (T(165) = 5.09 p < .001) used the Internet for health-related activities;
• 10.0% of the Omani encounters with physicians, and 4.0% of the German encounters (T(153) = 2.16 p = .043) were via email;
• 15.2% of the Omani encounters with physicians and 1.8% of the German encounters (T(154) = 4.47 p < .001) were via social media;
• 13.5% of the Omanis and 6.0% of the Germans brought information found online to their doctor's appointments (T(153) = 2.23 p = .027), and
• 7.6% of the Omanis and 1.7% of the Germans said that their doctors were likely to recommend medical websites or applications (T(159) = 2.71 p = .008).

### 3.3 Impact of demographics

Because patient demographics impact on e-patient activities, we evaluated the data in the above tables against the demographic data.

There were significant differences for age and origin for the knowledge of books (Oman: M = 34.02 SD = 7.25; Germany: M = 42.95 SD = 16.50; F(1, 79) = 11.44 p = .001), the knowledge of videos (Oman: M = 35.74 SD = 9.58; Germany: M = 43.97 SD = 14.76; F(1, 116) = 13.24 p < .001), the knowledge of magazines (Oman: M = 34.69 SD = 6.88; Germany: M = 47.68 SD = 15.93; F(1, 51) = 9.77 p = .003), and the usage of magazines (Oman: M = 32.89 SD = 5.65; Germany: M = 50.9 SD = 14.80; F(1, 28) = 12.36 p = .002).

There was a significant difference for age and origin in the usage of monitoring apps. Here, the Germans were older than the Omanis (Oman: M = 34.15 SD = 10.6; Germany: M = 59.60 SD = 15.19; F(1, 30) = 21.33 p < .001).

### 3.4 Impact of chronic vs. acute

There was no impact of chronic or acute pain on the use of the Internet for the Omanis, but the Germans who suffered from chronic conditions used videos more often than those with acute conditions (χ^2^(2) = 6.97, *P* = .031; chronic: N = 6; acute: N = 3). Further, the German chronic condition group also read more general references (χ^2^(2) = 9.60, *P* = .008; chronic: N = 7; acute: N = 1).

### 3.5 Patients' and doctors’ attitudes

Using questions based primarily on the study of doctors by Moick and Terlutter [48], we asked the patients about their and (from their perspective) their doctors' attitudes towards patients’ bringing information from the Internet to the consultation. Table 6 gives a summary of the results.

**Table 6:** Summary of responses to attitude statements.

Item	Germany (N = 83)		Oman (N = 93)		TOTAL (N = 176)		Statistics
	Mean	SD	Mean	SD	Mean	SD	
I think it is generally positive.	3.65	1.97	5.42	1.9	4.68	2.11	F (1, 162) = 33.54; *P* < .001
Doctors are prepared to correct wrong, incomplete and misunderstood information.	4.88	1.98	5.42	1.71	5.21	1.84	F (1, 154) = 3.20; *P* = .076
Doctors sometimes might feel as if they lost their authority and control.	3.46	2.16	3.47	1.97	3.47	2.04	F (1, 161) = 0.001; *P* = .975
I expect a more time-consuming patient visit than with un-informed patients.	3.02	1.81	5.10	1.92	4.25	2.13	F (1, 158) = 47.62; *P* < .001
The doctor-patient relationship will be improved by better communication	4.08	2.19	6.20	1.41	5.34	2.05	F (1, 159) = 56.02; *P* < .001
Doctors would be more likely to prescribe a desired medication than if the patients were uninformed	2.74	2.09	4.07	2.38	3.55	2.35	F (1, 156) = 12.87; *P* < .001

The Omanis agreed significantly more strongly that patients’ bringing information was positive, and that the physician-patient relationship would be improved. They also, however, felt more strongly that the patient visits would be more time-consuming, and that informed patients would more easily get a desired medication.

Among the Germans, age was correlated with patient contact taking more time (r = 0.345 p = .005), doctor-patient relationships improving because of better communication (r = 0.327 p = .008), and doctors being more likely to prescribe a desired medication (r = 0.298 p = .019). However, no correlations were found for age for the Omanis or overall.

Among both Germans and Omanis, patients who were being treated for chronic conditions felt more strongly that patients’ bringing Internet information to the consultation was generally positive, more time-consuming, would result in better communication and changes in medication (See Table 7).

**Table 7:** Summary of responses to attitude statements for patient with chronic conditions.

Item	Germany		Oman		Statistics
	Mean	SD	Mean	SD	
I think it is generally positive.	3.84	1.72	5.66	1.79	F (1, 61) = 16.06; *P* < .001
I expect a more time-consuming patient visit than with un-informed patients.	3.09	1.89	5.60	1.84	F (1, 61) = 27.13; *P* < .001
The doctor-patient relationship will be improved by better communication	3.52	2.04	6.11	1.43	F (1, 59) = 33.86; *P* < .001
Doctors would be more likely to prescribe a desired medication than if the patients were uninformed	2.32	1.78	4.15	2.27	F (1, 58) = 10.51; *P* = .002

## 4 Discussion

This paper has studied e-patient activities and attitudes at two surgical sites in Germany and Oman. German e-patient activity has been studied before [35,39], but this is the first known study of e-patients in Oman. There were many similarities, as the patients engage in typical e-patient activities, and they perceive the benefits of doing so.

Overall, even where there were similarities between the two countries, the Omani patients had greater knowledge and usage of health-related Internet sites and apps, and were more likely to bring information from the internet to the consultation. Although a similar number of patients had heard of the term “e-patient”, and communicated electronically with their doctors, the Omanis had a far higher level of contact than the Germans, and their activity correlated with their being more positive about the impact of their bringing material from the Internet.

These differences are discussed in more detail below, in which we consider the impact of demographics, type of treatment, and the broader socio-cultural context.

### 4.1 Impact of age and gender

Literature has frequently shown negative correlations between age and Internet usage, email and social media in general [6] and e-patient activities in particular [14,24,26,29,30,34,50]. In this study, the Omanis were significantly younger than the Germans, and, when drilling down into the knowledge and usage of Internet sites and apps, we find significant age differences; these could go some way in explaining the e-patient activity differences between the two countries. If this is so, then it serves as an alert for the future, that e-patient activities are only going to grow.

One must be careful, however, not to over-estimate the impact of age on e-patient activities. Firstly, there are studies that indicate that the elderly frequently use the Internet for seeking health-related information, particularly as they become more concerned about their health [51,52]. Secondly, the figures from our research indicate that, while knowledge and usage of some categories is associated with younger e-patients (especially books and videos), this was not across the board; many of the categories do not show correlations and one (Magazines) shows the opposite.

Similarly, although historically, males have used the Internet more than females, when looking at e-patient figures, the situation is not as distinct. For many years, several studies have shown that females generally use the Internet for health-related searches more than males (frequently because females are primary caregivers in their family) [4,5,53].

### 4.2 Impact of type of treatment

The literature also indicates that e-patient activity involvement is greater among patients suffering predominantly from chronic rather than acute conditions, and many Internet sites are specifically aimed at chronic conditions [9,11,12,20,28,31]. In our sample, a greater percentage of the Omanis than the Germans were at the hospital for chronic treatment, and so this may impact on the results and increase the differences between the two countries.

On examining the figures in more detail, however, we see that this statement is true to a limited extent only: while having a chronic condition does tend to increase e-patient activity, this is not true for all activities.

### 4.3 The broader socio-cultural contexts

The socio-cultural differences between the two countries also need to be considered. The previously-cited recent study on Omani surgeons' use of the Internet [42] had referred to the possible influence of Oman's high consanguinity rate (52%) (of which 75% is to first cousins [54]), the Omani surgeon's use of personal mobile devices for professional work, and the increase in social media usage in Oman since the 2011 “Arab Spring” [[55], [56], [57], [58]]. These factors may combine to increase electronic interaction between patient and physician.

In addition, physician-patient email communication was low 10–15 years ago [10], and has since increased; similarly, patients’ use of social media has previously primarily been in informal patient communities [11,13,59], but the patterns among Omanis may be an early indication of a shift towards increased social media interaction between patients and physicians. This is a possible area for future research, and it will have implications for medical education: just as medical students have required training on the appropriate use of email with e-patients [3], so too will they require training on appropriate social media interaction, especially because social media is far more intrusive.

Irrespective of the reasons for the differences between the two countries, the electronic patient engagement levels lead one to predict that the result should be a potential for positive health outcomes, as has been found in the much of the literature [35,60,61].

### 4.4 Limitations

This study was conducted at two sites only, and focused on patients’ interactions with surgeons. Further studies would lead to stronger generalisations, although it should be noted that Oman has few surgical centres and only two medical schools. Further, because this was the first study of e-patients in Oman, there were no controls on age or type of visitation. (Already, the focus on surgery was a control). While this is a limitation, it does serve to highlight the fact that the patterns of e-patient activities in Oman are impacted upon in a way that is similar to the impacts described in other literature. Because of the demographic and other influences, however, future studies could apply more controls in order to obtain a more nuanced understanding of e-patient activities.

## 5 Conclusion

In this study of e-patient activities at two surgical centres in Germany in Oman, we found typical e-patient activities, such as patients bringing information from the Internet and engaging electronically with their doctors. Overall, there appeared to be greater engagement by the Omani patients, especially in electronic communication with their doctors; some of these differences may be explained by age and type of condition, although the socio-cultural context also appears to have influenced use and attitude patterns. The broader implications appear to be chiefly that surgeons should be aware of e-patients’ growing use of the Internet, including the possible demand for interaction through social media, and that this will impact on healthcare delivery. More broadly, medical education, particularly Continuing Professional Development, should directly address the topic of working with the e-patient.

## Provenance and peer review

Not commissioned, Editor reviewed.

## Ethics

Ethics approval was obtained from the Sultan Qaboos University College of Medicine Research Ethics Committee (MREC#1628) and from the University of Tübingen's Medical Ethics Committee (No. 001/2018BO2).

## Informed consent

Informed consent was obtained from all individual participants included in the study.

## Disclosure of funding

We acknowledge support by 10.13039/501100001659Deutsche Forschungsgemeinschaft and 10.13039/501100002345Open Access Publishing Fund of University of Tuebingen.

## Data sharing statement

The dataset used and analysed during the current study are available from the corresponding author on reasonable request.

## CRediT authorship contribution statement

**Ken Masters:** Writing - original draft, Conceptualization, Methodology. **Teresa Loda:** Conceptualization, Formal analysis, Methodology. **Rashid Al-Abri:** Investigation, Supervision. **Jonas Johannink:** Investigation, Supervision. **Anne Herrmann-Werner:** Writing - review & editing, Conceptualization, Methodology.

## Declaration of competing interest

The authors report no conflicts of interests, financial or otherwise.
