# Supplementary material for: Surgical patients’ use of, and attitudes towards, the internet for e-patient activities in Germany and Oman
Source: Ann Med Surg (Lond). 2020 Jun 3;55:287–93. doi: 10.1016/j.amsu.2020.05.022 (PMC7287187; doi:10.1016/j.amsu.2020.05.022)
Supplement: Supplementary file 2 — Multimedia component 2 [file mmc2.pdf]

Patient Questionnaire

Dear Patient,
Please do not refer to information given to you by another doctor. This questionnaire focuses information in the Internet.

1. My Age is ____________ 2. My Gender is ☐ female ☐ male ☐ prefer not to say

3. How many hours a day do you use the Internet?

☐ 0 ☐ 1-2 ☐ 3-4 ☐ 5-6 ☐ 7-8 ☐ 9-10 ☐ >10

4. What percentage of this time do you use it on health-related activities? Please mark the appropriate number (in %) by circling the number.

0% 5 10 15 20 25 30 35 40 45 50 55 60 65 70 75 80 85 90 95 100%

5. I **know of** this medical/health web site type:

☐ Online books (e.g. Google books, Thieme E-Book)
☐ Online videos (e.g. youtube, medtube, Visite)
☐ General references (e.g. Up to date, AMWF-Guidelines, Wikipedia, Medscape, WebMD)
☐ Networking sites (e.g. EbM-Network, Facebook groups)
☐ Official/ institutional bodies (e.g. Ärztliches Zentrum für Qualität in der Medizin, DKFZ)
☐ Online databases (e.g. PubMed, Google Scholar, MediSuch)
☐ Journals (e.g. British Medical Journal, Lancet, NEMJ, JAMA, Annals of Surgery, British journal of surgery)
☐ Magazines (e.g. Apothekenumschau, Focus Gesundheit, Men’s Health)

6. I **use** this website type **at least once per month:**

☐ Online books (e.g. Google books, Thieme E-Book)
☐ Online videos (e.g. youtube, medtube, Visite)
☐ General references (e.g. Up to date, AMWF-Guidelines, Wikipedia, Medscape, WebMD)
☐ Networking sites (e.g. EbM-Network, Facebook groups)
☐ Official/ institutional bodies (e.g. Ärztliches Zentrum für Qualität in der Medizin, DKFZ)
☐ Online databases (e.g. PubMed, Google Scholar, MediSuch)
☐ Journals (e.g. British Medical Journal, The Lancet, NEMJ, JAMA, Annals of Surgery, British journal of surgery)
☐ Magazines (e.g. Apothekenumschau, Focus Gesundheit, Men’s Health)

7. I **know** of this medical/health app type:

☐ Monitoring (e.g. Blood Pressure, Diabetes, Sleep)
☐ Information (e.g. Wikipedia, Flexicon, Net-doktor, Focus Gesundheit)
☐ Tools (e.g.BMI-Calculator, Runtastic, Calories counter)
☐ Videos (e.g. Youtube)

8. I **use** this app type **at least once per month**:

☐ Monitoring (e.g. Blood Pressure, Diabetes, Sleep)

☐ Information (e.g. Wikipedia, Flexicon, Net-doktor, Focus Gesundheit)
☐ Tools (e.g.BMI-Calculator, Runtastic, Calories counter)
☐ Videos (e.g. Youtube)

9. Have you heard of the term “e-patient” (to refer to a patient who looks up medical information on the Internet)

☐ Yes ☐ No

10. Do you interact with your doctor via email or through social media?

☐ Yes ☐ No

If yes, which of these

☐ Email ☐ Twitter
☐ Facebook ☐

10a) In how many cases of all your doctor-patient-contacts do you interact via email with your doctor? Please mark the appropriate number (in %) by circling the number.

0% 5 10 15 20 25 30 35 40 45 50 55 60 65 70 75 80 85 90 95 100%

10b) In how many cases of all your doctor-patient-contacts do you interact through social media with your doctor? Please mark the appropriate number (in %) by circling the number.

0% 5 10 15 20 25 30 35 40 45 50 55 60 65 70 75 80 85 90 95 100%

11. To how many of your visits to a doctor do you bring material with you or refer to information that you found on the Internet? Please mark the appropriate number (in %) by circling the number.

0% 5 10 15 20 25 30 35 40 45 50 55 60 65 70 75 80 85 90 95 100%

12. When you think of all your visits to a doctor in the past 12 months, how many times have you been recommended a website or app by a doctor? Please mark the appropriate number (in %) by circling the number.

0% 5 10 15 20 25 30 35 40 45 50 55 60 65 70 75 80 85 90 95 100%

13. If you marked more than 0%, name one website or app that you have been recommended

____

14a) Today's visit at the surgery is due to which main complaint?

Choice of:	☐ Chronic condition	☐ Acute condition

14b) If you are visiting due to an acute condition today, do you also suffer from a chronic condition?

☐ Yes ☐ No

15. Please indicate the degree to which you agree with the statements below:

If a patient brought some health-related information to a consultation…..

a) … I think it is generally positive.

I absolutely disagree	**1**	**2**	**3**	**4**	**5**	**6**	**7**	I absolutely agree

b) … doctors are prepared to correct wrong, incomplete and misunderstood information.

I absolutely disagree	**1**	**2**	**3**	**4**	**5**	**6**	**7**	I absolutely agree

c) … doctors sometimes might feel as if they lost their authority and control.

I absolutely disagree	**1**	**2**	**3**	**4**	**5**	**6**	**7**	I absolutely agree

d) … I expect a more time-consuming patient visit than with uninformed patients

I absolutely disagree	**1**	**2**	**3**	**4**	**5**	**6**	**7**	I absolutely agree

e) … the doctor-patient relationship will be improved by better communication.

I absolutely disagree	**1**	**2**	**3**	**4**	**5**	**6**	**7**	I absolutely agree

f) … doctors are more likely to prescribe a desired medication than if the patients were uninformed.

I absolutely disagree	**1**	**2**	**3**	**4**	**5**	**6**	**7**	I absolutely agree
